# Low dose docosahexaenoic acid protects normal colonic epithelial cells from araC toxicity

**DOI:** 10.1186/1471-2210-5-7

**Published:** 2005-03-23

**Authors:** Ming C Cha, Angela Lin, Kelly A Meckling

**Affiliations:** 1 Department of Human Biology and Nutritional Sciences University of Guelph, Guelph, Ontario, N1G 2W1, Canada

## Abstract

**Background:**

The nucleoside analogue arabinosylcytosine (araC) has been used for many years in the treatment of acute leukemia. Evidence in the literature suggests that araC may inhibit the growth of human colon carcinoma cell lines as well. Because araC action interferes with normal nucleoside metabolism, it is highly toxic to a number of normal cell types including bone marrow and intestinal mucosa cells. Here we investigate whether the omega-3 fatty acid docosahexaenoic acid (DHA) could selectively target araC toxicity toward colonic tumor cells while protecting the normal cells *in vitro*.

**Results:**

Cultures of normal rat colonic epithelial cells (4D/WT) and those transformed by *v-src *(D/v-src) were supplemented with graded concentrations of DHA or arachidonic acid (AA) alone or in combination with araC. AraC was only 1.6 fold more toxic to D/v-src than 4D/WT in cultures without added fatty acids. Supplementing with as little as 3 μM of either AA or DHA increased araC toxicity by more than 30-fold in the tumorigenic cells. The toxic effect of araC on the normal cells was also increased by the fatty acid supplementation. IC_50 _values were decreased 1.7 fold by DHA in the 4D/WT cells but a more than 7-fold decrease was observed during AA supplementation. As a result, the therapeutic index of araC (IC_50 _normal/IC_50 _tumor) was more than 3-fold higher in the DHA than the AA supplemented cells. The expression of protein kinase C isoform epsilon was decreased in AA alone supplemented D/v-src cultures but in combination with araC decreased only in DHA supplemented 4D/WT cells.

**Conclusion:**

Low dose DHA supplementation may enhance araC chemotherapy in colon cancer while protecting normal tissues, possibly through control of PKC signalling pathways.

## Background

Colorectal cancer is the second most common cause of death among men and women in North America [1]. This disease may evolve from genetic alterations in protooncogenes or tumor suppressor genes. There has also been evidence indicating dietary factors, such as fat and fiber, affect several stages of colon carcinogenesis. Japanese have a lower incidence of colon cancer as compared to North Americans, which could be partially attributed to higher amounts of n-3 fatty acids and low amounts of saturated, monounsaturated and n-6 fatty acids in their diets [2].

Arabinosylcytosine (araC) has been used for many years in the treatment of acute leukemia. Some later studies have suggested that araC and the related drug gemcitabine may be useful in inhibiting the growth of human colon carcinoma cell lines [3]. Because araC action interferes with normal nucleoside metabolism, a number of normal cell types particularly dependent on salvage of nucleosides, are extremely sensitive to araC; these include the bone marrow and intestinal mucosa. In patients with leukemias and non-Hodgkins lymphoma treated with araC, gastrointestinal and hematopoietic toxicities predominate and frequently limit dose escalation [4].

We have previously demonstrated that docosahexaenoic acid (DHA) can enhance araC and doxorubicin toxicity in leukemia cells and araC toxicity in transformed rat fibroblasts *in vitro *[5,6]. More recently, we showed that at least part of this differentially selectivity was due to effects of DHA on two metabolic enzymes cytidine kinase and cytidine deaminase [7]. *In vivo *we showed that DHA had beneficial effects on both the bone marrow compartment and gastrointestinal tract of tumor-bearing, araC treated rats [8]. We also demonstrated that a low dose of dietary DHA supplementation could prolong the life span and limit the occurrence of toxicity in L1210 leukemic mice [9]. The current study was designed to investigate whether low dose DHA could selectively target araC toxicity toward rat colon tumor cells *in vitro*.

Studies show that araC engages in an array of signaling events including activation of protein kinase C (PKC) [10]. Essential fatty acids of the omega-3 and omega-6 series are also known to have effects on cell membrane signaling which includes PKC [11,12]. Because both araC and DHA could potentially modulate cell death and drug toxicity in overlapping pathways, we also examined the expression of various PKC isoforms during fatty acid supplementation and drug treatment.

## Results

Using the sulforhodamine dye binding assay the IC_50 _values for arachidonic acid (AA), DHA, and araC were examined in the normal colonic epithelial cell line 4D/WT and in the v-src transformed variant, D/v-src (Table 1.). AA and DHA had approximately the same toxicity profile in both cell types and showed 5-7-fold higher toxicity in the tumorigenic versus the normal epithelial cells. The IC_50 _value for araC in 4D/WT was 67 μM and only slightly lower in the D/v-src cells at 42 μM. Pre-incubating 4D/WT cells with 3 μM DHA (well below the IC_50 _values for either cell type) only slightly increased araC toxicity (1.7-fold) while pre-incubation with 3 μM AA increased araC toxicity 7-fold in the normal 4D/WT cells. Pre-incubation of the tumorigenic cell line, D/v-src with either 3 μM DHA or AA resulted in a dramatic 40-fold increase in araC toxicity. Therefore the improvement in therapeutic index is 30-fold in the presence of 3 μM DHA (p < 0.03). Toxicity of other fatty acids (saturated, unsaturated, series n-9, n-6 and n-3) either alone or in combination with araC were not different between normal and tumor cells in culture (data not shown).

**Table 1:** Toxicity of fatty acids or araC alone and in combination on normal (4D/WT) and transformed (D/v-src) rat colonic epithelial cells^1^

	DHA (n = 3)	AA (n = 3)	AraC (n = 3)	AA-araC (n = 3)	DHA-araC (n = 4)
	IC_50 _μM				
4D/WT cells	87.8 ± 10.2	91.1 ± 18.4	67.4 ± 11.1	9.3 ± 1.4	39.1 ± 10.4
D/v-src cells	14.9 ± 5.9*	12.2 ± 5.0*	41.8 ± 2.8	1.0 ± 0.6	1.3 ± 0.5*

^1^For the drug and fatty acid toxicity assays, cells were cultured in media supplemented with graded concentrations of araC or the fatty acids for 24 hrs. For the fatty acid supplemented drug toxicity assays, cells were treated with 3 μM DHA or AA for 24 hrs. Cells were then cultured in media supplemented with DHA or AA and graded concentrations of araC for an additional 24 hrs. Values are means ± SEM with sample sizes indicated in parenthesis. Values followed by * are significantly different from the values within the same column (p < 0.05).

**Figure 1:**
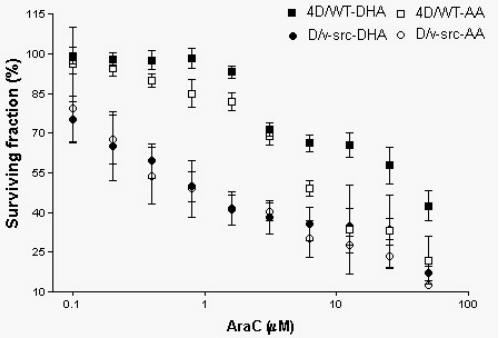
Survival curves for cells cultured in media supplemented with DHA or AA and treated with graded concentrations of araC. 4D/WT and D/v-src cells were cultured in media supplemented with 3 μM DHA or AA for 24 h. Fresh media was added to include both fatty acids and graded levels of araC for an additional 24 h. Cell viability was determined by the SRB dye binding assay by comparing to control cells not treated with fatty acid or araC. Values are means ± SEM of 3 – 4 separate experiments. Significant differences were detected between tumor and normal cells at all concentrations of drug tested up to 10 μM araC, beyond which only normal cells co-supplemented with DHA grew significantly better than all other cell cultures. DHA and AA supplemented tumor cell cultures were not statistically different from each other at any araC concentration tested. DHA and AA supplemented normal cells were not significantly different at 0.1, 0.2 and 3 μM araC. AraC was significantly more toxic to the AA supplemented normal cells compared to DHA supplemented normal cells at all other concentrations of drug.

The survival curves in araC treated cultures with or without fatty acid supplementation are shown in Figure 1. The survival rates were not different between the normal and the transformed cells at very low araC concentrations (0.1 and 0.2 μM). At 0.4 μM araC the surviving fraction of normal cells is statistically higher for 4D/WT cells in either DHA or AA supplemented media (> 97%) compared to D/v-src cells (<60%). At about 6 μM araC there is no therapeutic benefit of AA treatment given that the level of kill is equivalent between 4D/WT and D/v-src cells at this point in the dose response curve. However, in DHA-treated 4D/WT cells, the survival curves were statistically higher than D/v-src cells at all araC concentrations and never dropped below 50% over the range of concentrations used in this experiment.

**Figure 2:**
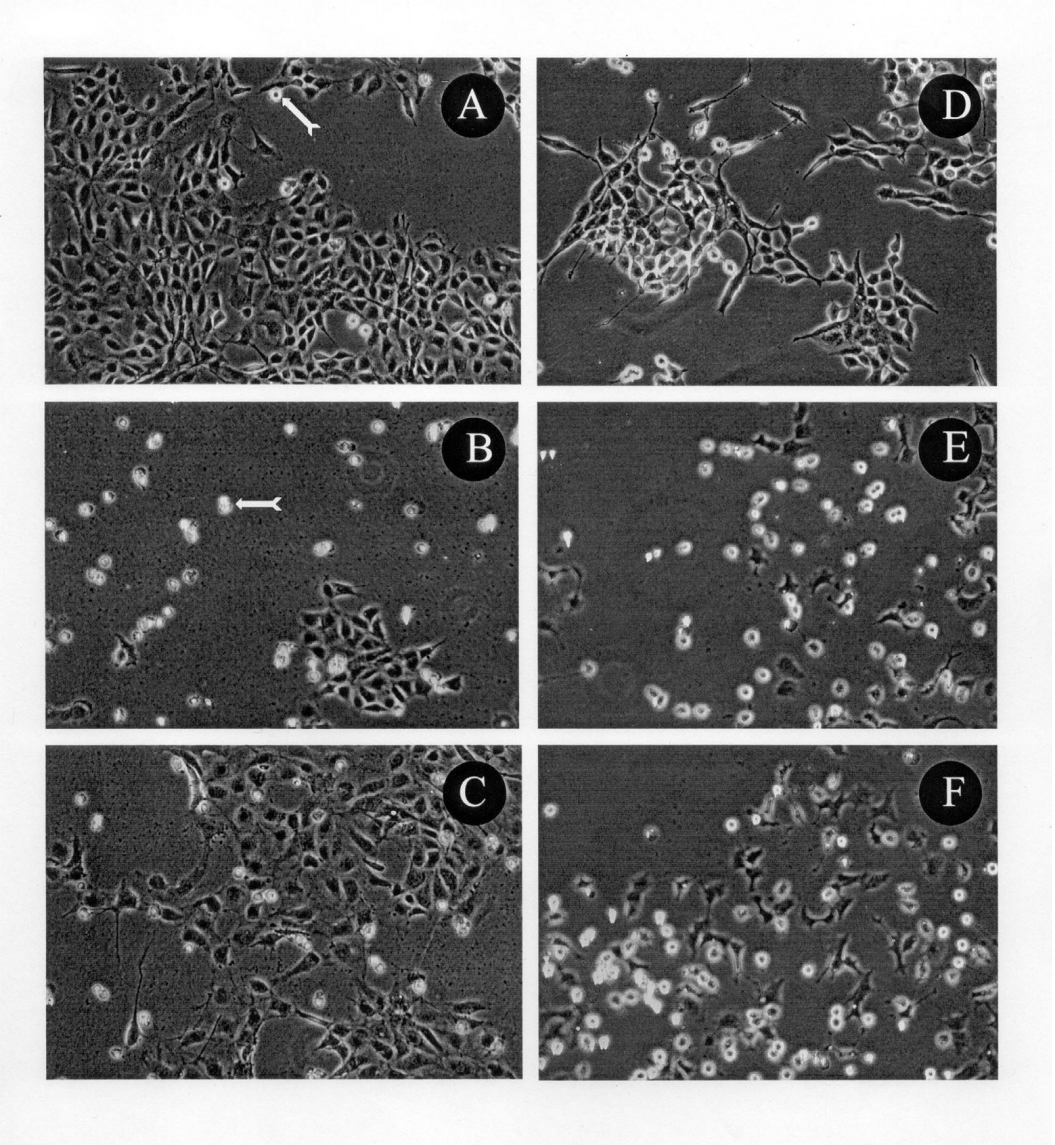
The effect of araC on the morphology of 4D/WT and D/v-src cells treated with or without 3 μM AA or DHA. 4D/WT cells (**A**, **B**, **C**) or D/v-src cells (**D**, **E**, **F**) were cultured in medium supplemented with 3 μM AA (**B**, **E**) or DHA (**C**, **F**) or a medium without the fatty acid supplementation (**A**, **D**) for 24 h. Cells were then treated with either 10 μM (4D/WT cells, **A**, **B**, **C**) or 2 μM (D/v-src cells, **D**, **E**, **F**) araC for an additional 24 h. Dead cells are indicated by the arrow signs. All photographs are at 200× magnification.

The effect of araC on the morphology of 4D/WT and D/v-src cells treated with or without DHA or AA is shown in Figure 2. AraC at 10 μM, killed only a few 4D/WT cells growing in 10% FBS containing medium (panel A). More 4D/WT cells were killed when cultures were supplemented with DHA (panel C), however less than half of the cells survived when AA replaced DHA in the araC treated cultures (panel B). In contrast, 2 μM araC caused potent killing in D/v-src cultures supplemented with either AA (panel E) or DHA (panel F) compared to araC only treated cultures (panel D).

**Figure 3:**
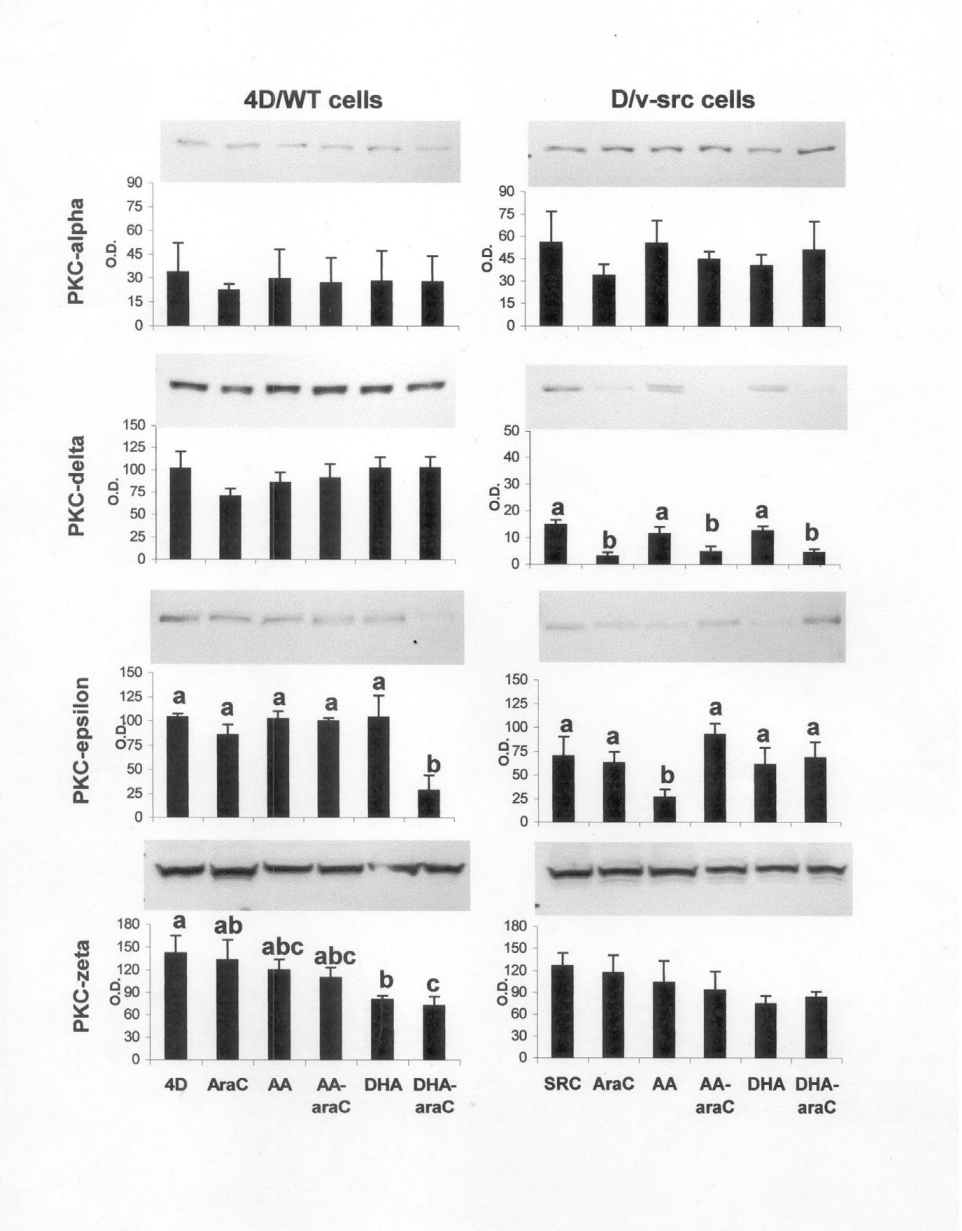
PKC isoform expression as determined by Western blot. 4D/WT and D/v-src cells were cultured in media supplemented with 3 μM DHA or AA for 24 h. Fresh media with/without the fatty acids and 10μM araC was added. Cell lysates containing 30 μg protein/lane were loaded and separated on 10% SDS-PAGE gels. Gels were transferred and probed as described in the Materials and Methods. Densitometric values are means ± SEM of 3 – 4 separated experiments. Bars with different letters within the same PKC isoform and cell type are significantly different (Fisher's protected LSD test).

The expression of the PKC isozymes α, δ, ε and ζ in 4D/Wt and D/v-src cells treated with or without araC or the fatty acids or a combination of the fatty acids and araC are shown in Figure 3. PKC δ expression was higher in normal colonic epithelial cells than in the transformed variant. Treatment with araC decreased PKC δ expression in the D/v-src cells in both FBS only and DHA/AA supplemented conditions. Less PKC ε was detected in the AA supplemented D/v-src compared to all other treatments. Densitometry and statistical analysis revealed an interaction between araC and fatty acid treatment in both the 4D/WT and D/v-src cells. In the expression patterns of PKC isoforms ε and ζ, the expression of both PKC isoforms was decreased by araC only when DHA was supplemented. There was no statistically significant change in PKC α expression under any treatment conditions.

## Discussion

There is considerable evidence that nutrition and lifestyle factors have major impact on the early stages of carcinogenesis in the colon and other tissues. What is less clear is the role, if any, for nutritional supplementation as part of an adjuvant therapeutic strategy in colon cancer treatment. Given that colon cancer is particularly resistant to existing chemotherapeutic drugs, and at best an increase of 20% in five-year survival rate is achieved with combination 5-fluorouracil (5-FU) and levamisole/leucovorin, improvements in drug toxicity/selectivity would certainly be helpful.

Nucleoside chemotherapeutic drugs including araC, gemcitabine and 5-FU all have intracellular targets and interfere with the metabolism of normal nucleosides to disrupt cellular growth. Tissues with high turnover rates such as bone marrow and intestinal mucosa are particular sensitive to those drugs. The present study demonstrated that low dose DHA substantially enhanced araC's ability to kill tumorigenic colonic epithelial cells while only marginally increasing toxicity toward the normal mucosal cell line. While AA also had sensitizing activity, it was much less selective than was DHA. While the concentration of DHA in normal FBS, or in the blood of individuals consuming habitual North American diets is very low, we and others have shown that levels as high as 100 μM can be achieved in blood plasma through dietary supplementation of mouse diets [9]. In several *in vitro *and *in vivo *systems we, and others, have demonstrated that long chain polyunsaturated fatty acids, particularly those found in fish oils, can enhance tumor kill by chemotherapeutic drugs and protect normal tissues [5,8,13]. In leukemia treatment, where araC is often used in combination therapy, the plasma levels reach up to 2 μM during high dose or low dose infusions. The observation here, that distinct differences in toxicity were observed between 0.1 and 1 μM, supports the idea that DHA will improve the clinical efficacy of araC at therapeutically relevant doses.

Enterocytes and colonocytes express many PKC isoforms including α, β_I_, β_II_, δ, ε, η, θ, ζ, and ι. most of which are activated in the post-mitotic compartment. Colon tumors from both humans and rats tend to show decreased abundance of α, β_I_, δ, ε, ζ and/or η. Perlletti and co-workers have demonstrated a lower PKC δ expression in rat colonic epithelial cells [14]. Consistent with those previous reports, we showed significantly lower expression of PKC δ in D/v-src cells as compared to 4D/WT cells. Over-expression of δ in CaCo-2 human colon tumor cells has been shown to decreased growth [15] and at least partially reverses the malignant phenotype of *src *transformed rat colonic epithelial cells [14]. PKC is an important modulator of araC toxicity [10,16] and a global reduction in PKC expression correlates with increased araC cytotoxicity [16]. Our study showed a potent down-regulation of PKC δ with araC treatment in tumor (CLD/src) but not in normal cells (CLD/WT), which may be related to the higher cytotoxity of araC to the transformed cells, in accordance with the previous report.

Chapkin's group has shown that dietary fat type can modulate PKC isozyme expression patterns in the colon [17]. A recent study by Mirnikjoo [18] showed that EPA and DHA, but not AA, were active inhibitors of the catalytic domains of several kinases including PKC. Nair and coworkers [19] showed similar selectivity of n-3 fatty acids to modulate PKC activity and subcellular redistribution in cardiac myocytes. In the present study, PKC ζ expression was decreased by the presence of DHA but not by AA in the normal colon cells with or without araC treatment, suggesting DHA can modify the PKC expression in normal cells. However, our results didn't show a significant effect of DHA on the levels of this PKC isozyme expressed in the transformed cells, suggesting an interaction between the cell type and the effect of DHA on PKC expression. PKC ε content was also influenced by the fatty acids and drug in a complicated way. The decreased expression of ε in the D/v-src cells in the presence of AA was not observed when cells were co-treated with araC. In the 4D/WT cells, on the other hand, AA and DHA alone had no effect on PKCε expression but the DHA-araC combination did down regulate this isozyme. If one looks for correlations between the toxicity profiles and PKC expression profiles, an ability to down regulate PKCε expression in 4D/WT is correlated with lower toxicity of araC. An obvious question is how this might be achieved at a molecular level, particularly since individually DHA and araC had no effect on expression, and AA had no combinatorial effect. Though there does not appear to be a single dominant effect of one PKC isoform over another, it could be the combination pattern of expression of the various PKCs that is critical in determining the final response. For example, the high expression of PKC ε and low expression of PKC δ in D/v-src may be a marker of susceptibility. Further experiments are required to determine the molecular nature of these interacting signalling pathways.

## Conclusion

In summary, the present study demonstrated that low dose DHA or AA supplementation synergistically interacts with araC to achieve therapeutic gain against the colon tumor cells. Because DHA, but not AA showed tumor cell selectivity, this suggests that dietary supplementation protocols including DHA with araC, gemcitabine or other drugs should be tested in pre-clinical models of colon cancer.

## Methods

### Cell culture

The cell lines used in the present research were a generous gift from Dr. Susan E. Pories, New England Deaconess Hospital and Harvard Medical School. These cells developed by Pories et al [20], consist of two mycoplasma-negative rat colonic epithelial cell lines and the immortalized nontumorigenic cell line referred to as FRC TEX CL 4D (referred throughout the text as 4D/WT) and the derivative transformed cell line FRC TEX CL D/v-src (referred to as D/v-src), established by transfection of a v-src sequence into the 4D/WT parental line. D/v-src cells exhibit the neoplastic phenotype while 4D/WT do not display anchorage independent growth or form tumors *in vivo*. 4D/WT and D/v-src cells were cultured in Dulbecco's modified Eagle's medium with 10% fetal bovine serum (FBS), hydrocortisone (0.02 ug/mL), insulin (0.25 ug/mL), transferrin (0.12 ug/mL), polybrene (1 ug/mL) and penicillin-streptomycin (50 U/mL) in a humidified environment at 37°C in the presence of 10% CO_2_.

### Toxicity assays

IC_50 _values were determined for 4D/WT and D/v-src cells treated with or without DHA or AA or a combination of araC and the fatty acids by protein binding dye sulphorhodamine B (SRB) assay as we have previously described (5,6). DHA or AA as the free fatty acids were first bound to albumin in serum for 1 h at 37°C before diluting into culture media at 0–500 :M. For the drug (0–1 mM) or fatty acid toxicity assays, approximately 1000 cells/well were plated in 96-well plates and cultured for 24–48 h. Cells were allowed to adhere for a minimum of 6 h before the fatty acid or drug was added.

### PKC protein expression

Sub-confluent monolayers of 4D/WT and D/v-src cells were grown in media supplemented with or without 3 μM DHA or AA in 100mm plates for 24 h. Media was then replaced with fresh media ± fatty acid, and/or ± 10 μM araC and cultured for an additional 24 h. Cells were harvested, washed three times in cold phosphate buffer saline (PBS) and resuspended in a small volume of PBS containing protease inhibitor cocktail (Boehringer Mannheim). Samples were sonicated, 10 μL of lysate recovered for protein assay (Bio-Rad Laboratories, Inc., Hercules, CA), and an equal volume of 2× hot Laemmli sample buffer was added. Proteins (30 μg/well) were separated by SDS-PAGE on 10% acrylamide gels and transferred to nitrocellulose membrane on a semidry transfer apparatus. Membranes were blocked in 5% milk powder in Tris-buffered saline (TBS) and probed with the respective primary monoclonal antibody (Transduction Laboratories, Lexington, KY) at concentration range from 1:500 to 1:1500 dilution in 1% BSA. Membranes were washed and then probed with secondary antibodies conjugated to horseradish peroxidase diluted in 5% milk. Blots were developed using the enhanced chemiluminescence method (Amersham) and density of bands quantified by an imaging densitometer using Northern Eclipse software (Empix Imaging Inc, Missisaga, ON).

### Statistical analysis

Analysis was performed using SPSS general linear model program version 7.5 (SPSS Inc. Chicago, IL). The effect of treatment was determined by one-way ANOVA and Fisher's protected LSD post-hoc test with significance determined to be p < 0.05. For the cell viability analysis, data were log transformed to obtain a normal distribution and plotted and IC_50 _values determined using a CurveFit computer software.

## Authors' contributions

MC carried out the PKC protein expression assay and drafted the manuscript. AL carried out the toxicity assay. KM conceived of the study and participated in its coordination. All authors read and approved the final manuscript.
